# Supplementary material for: Signatures of genetic variation in human microRNAs point to processes of positive selection and population-specific disease risks
Source: Hum Genet. 2022 Mar 6;141(10):1673–93. doi: 10.1007/s00439-021-02423-8 (PMC9522702; doi:10.1007/s00439-021-02423-8)
Supplement: Supplementary file 1 — Supplementary file1 (PPTX 1343 KB) [file 439_2021_2423_MOESM1_ESM.pptx]

## Slide 1
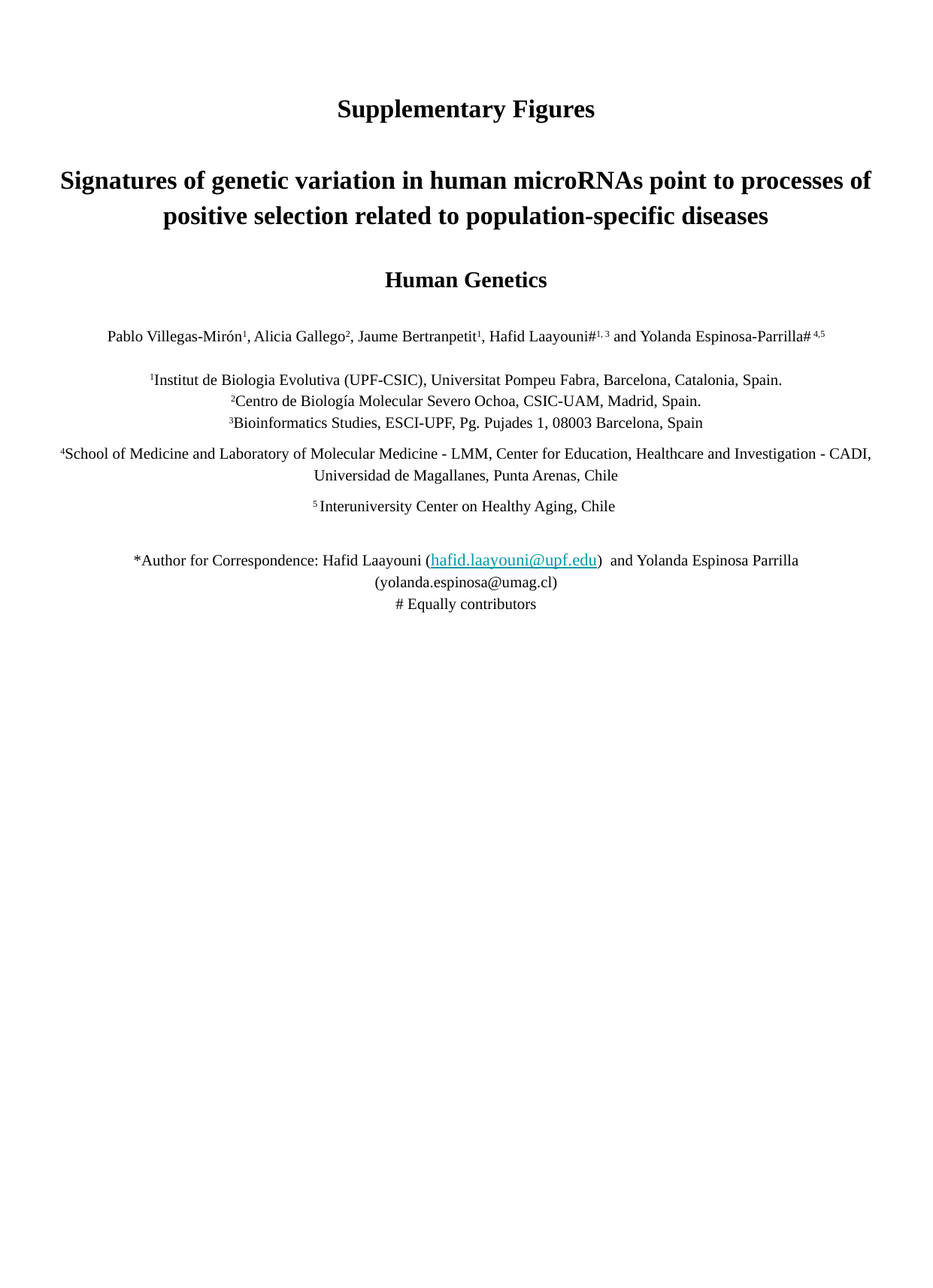

# Supplementary Figures
Signatures of genetic variation in human microRNAs point to processes of positive selection related to population-specific diseases
Human Genetics
Pablo Villegas-Mirón1, Alicia Gallego2, Jaume Bertranpetit1, Hafid Laayouni#1, 3 and Yolanda Espinosa-Parrilla# 4,5
1Institut de Biologia Evolutiva (UPF-CSIC), Universitat Pompeu Fabra, Barcelona, Catalonia, Spain.
2Centro de Biología Molecular Severo Ochoa, CSIC-UAM, Madrid, Spain.
3Bioinformatics Studies, ESCI-UPF, Pg. Pujades 1, 08003 Barcelona, Spain
4School of Medicine and Laboratory of Molecular Medicine - LMM, Center for Education, Healthcare and Investigation - CADI, Universidad de Magallanes, Punta Arenas, Chile
5 Interuniversity Center on Healthy Aging, Chile
*Author for Correspondence: Hafid Laayouni (hafid.laayouni@upf.edu) and Yolanda Espinosa Parrilla (yolanda.espinosa@umag.cl)
# Equally contributors

## Slide 2
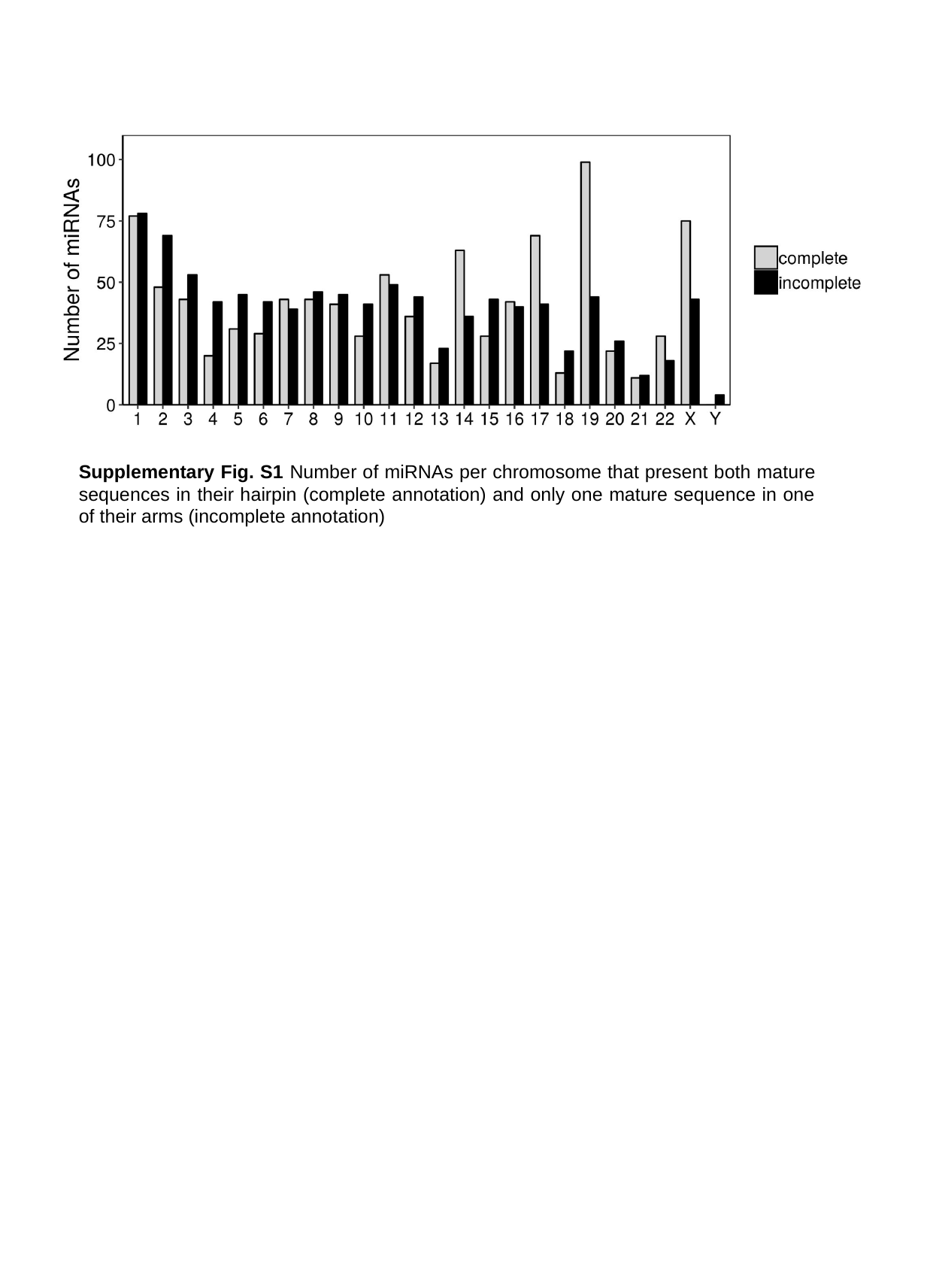

Supplementary Fig. S1 Number of miRNAs per chromosome that present both mature sequences in their hairpin (complete annotation) and only one mature sequence in one of their arms (incomplete annotation)

## Slide 3
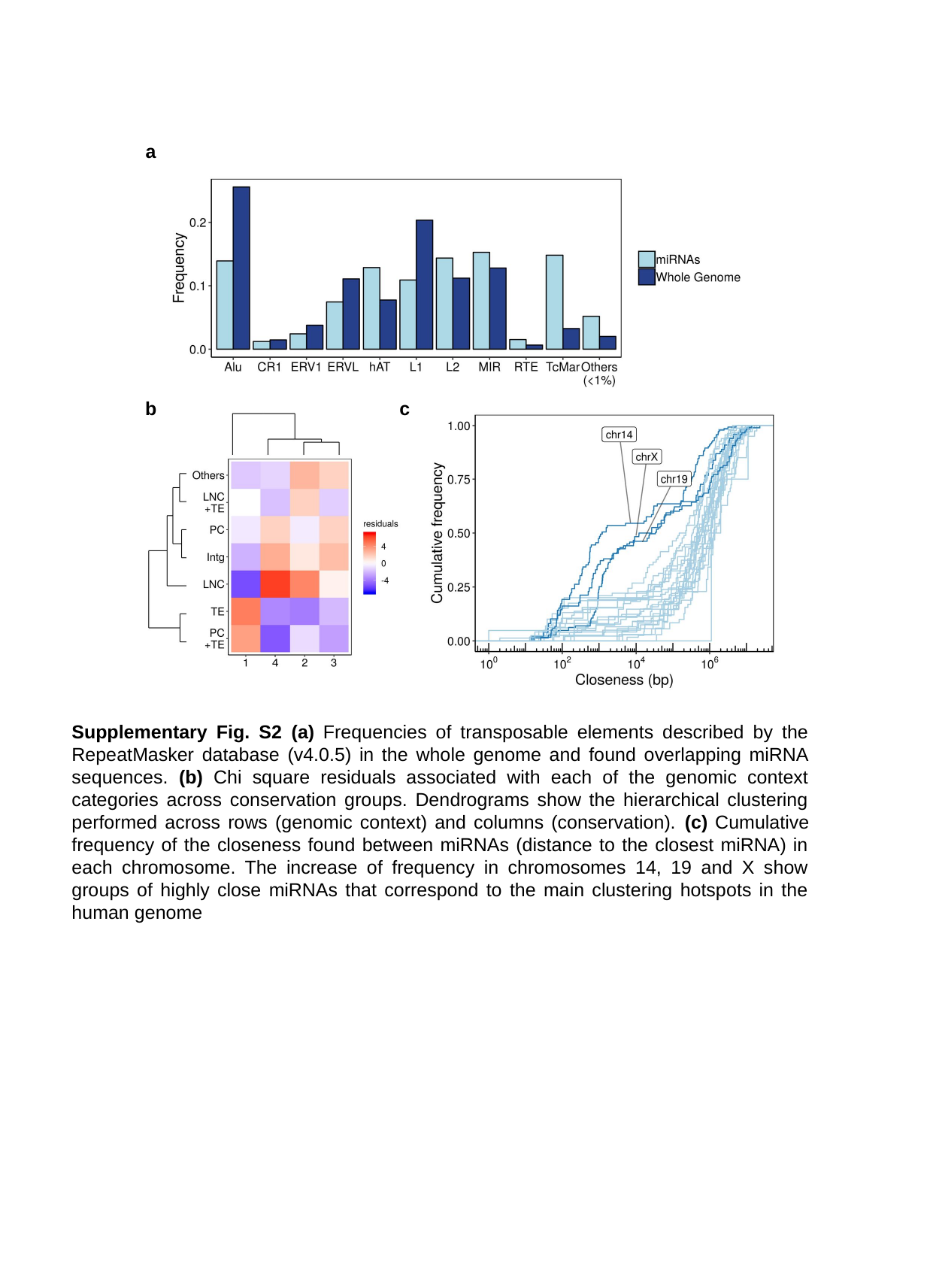

a
b
c
Supplementary Fig. S2 (a) Frequencies of transposable elements described by the RepeatMasker database (v4.0.5) in the whole genome and found overlapping miRNA sequences. (b) Chi square residuals associated with each of the genomic context categories across conservation groups. Dendrograms show the hierarchical clustering performed across rows (genomic context) and columns (conservation). (c) Cumulative frequency of the closeness found between miRNAs (distance to the closest miRNA) in each chromosome. The increase of frequency in chromosomes 14, 19 and X show groups of highly close miRNAs that correspond to the main clustering hotspots in the human genome

## Slide 4
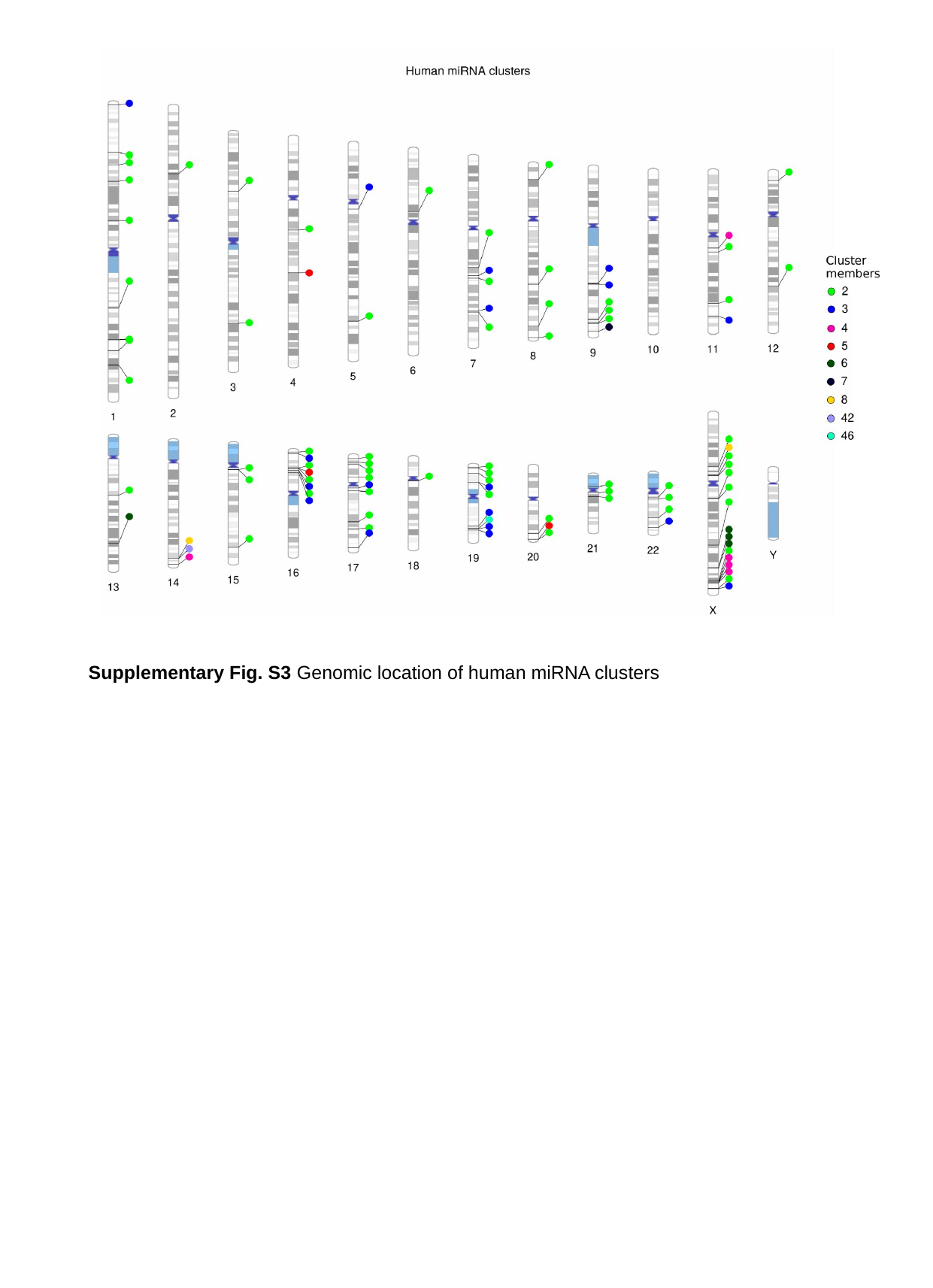

Supplementary Fig. S3 Genomic location of human miRNA clusters

## Slide 5
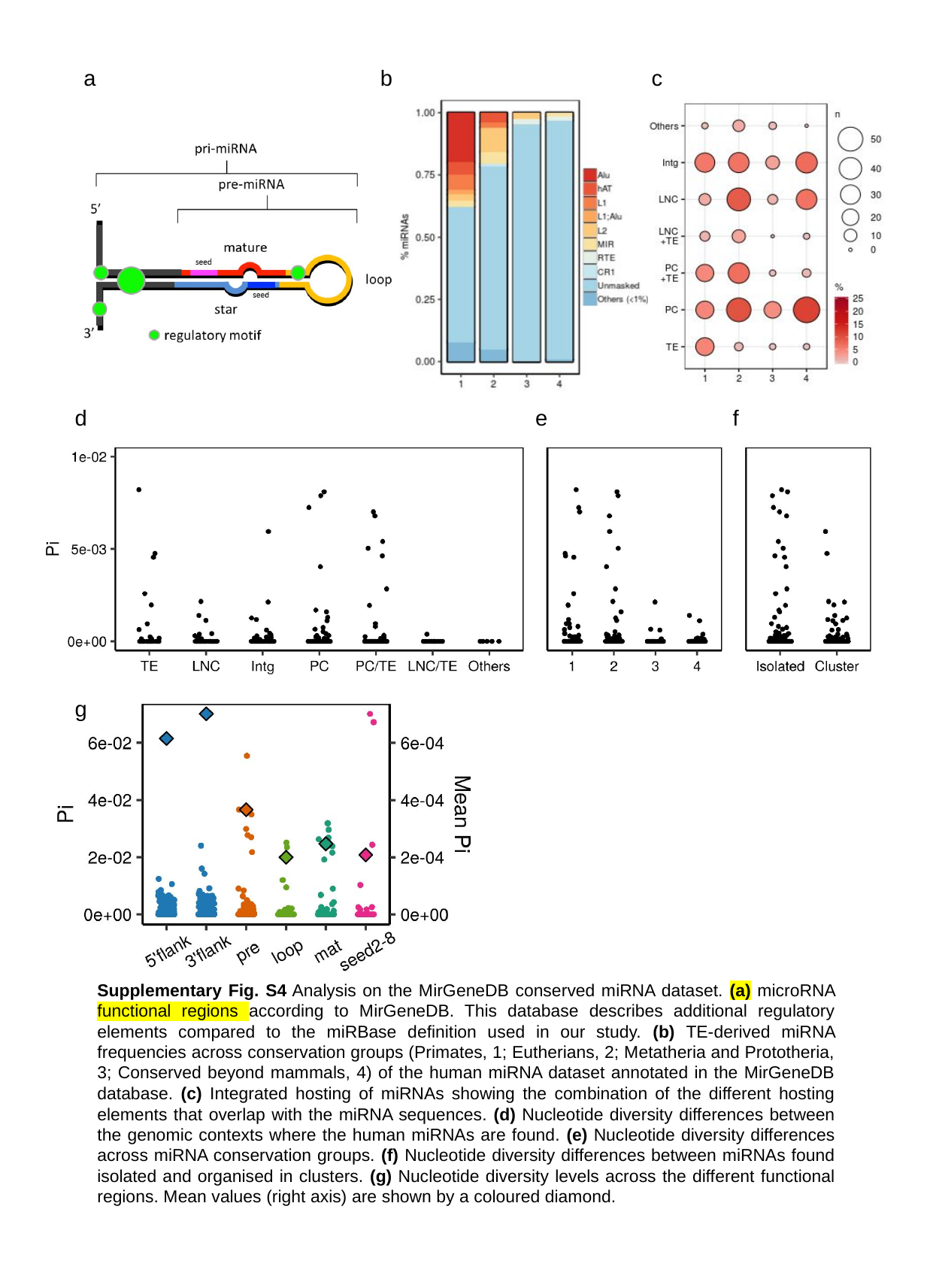

a
b
c
d
e
f
g
Supplementary Fig. S4 Analysis on the MirGeneDB conserved miRNA dataset. (a) microRNA functional regions according to MirGeneDB. This database describes additional regulatory elements compared to the miRBase definition used in our study. (b) TE-derived miRNA frequencies across conservation groups (Primates, 1; Eutherians, 2; Metatheria and Prototheria, 3; Conserved beyond mammals, 4) of the human miRNA dataset annotated in the MirGeneDB database. (c) Integrated hosting of miRNAs showing the combination of the different hosting elements that overlap with the miRNA sequences. (d) Nucleotide diversity differences between the genomic contexts where the human miRNAs are found. (e) Nucleotide diversity differences across miRNA conservation groups. (f) Nucleotide diversity differences between miRNAs found isolated and organised in clusters. (g) Nucleotide diversity levels across the different functional regions. Mean values (right axis) are shown by a coloured diamond.

## Slide 6
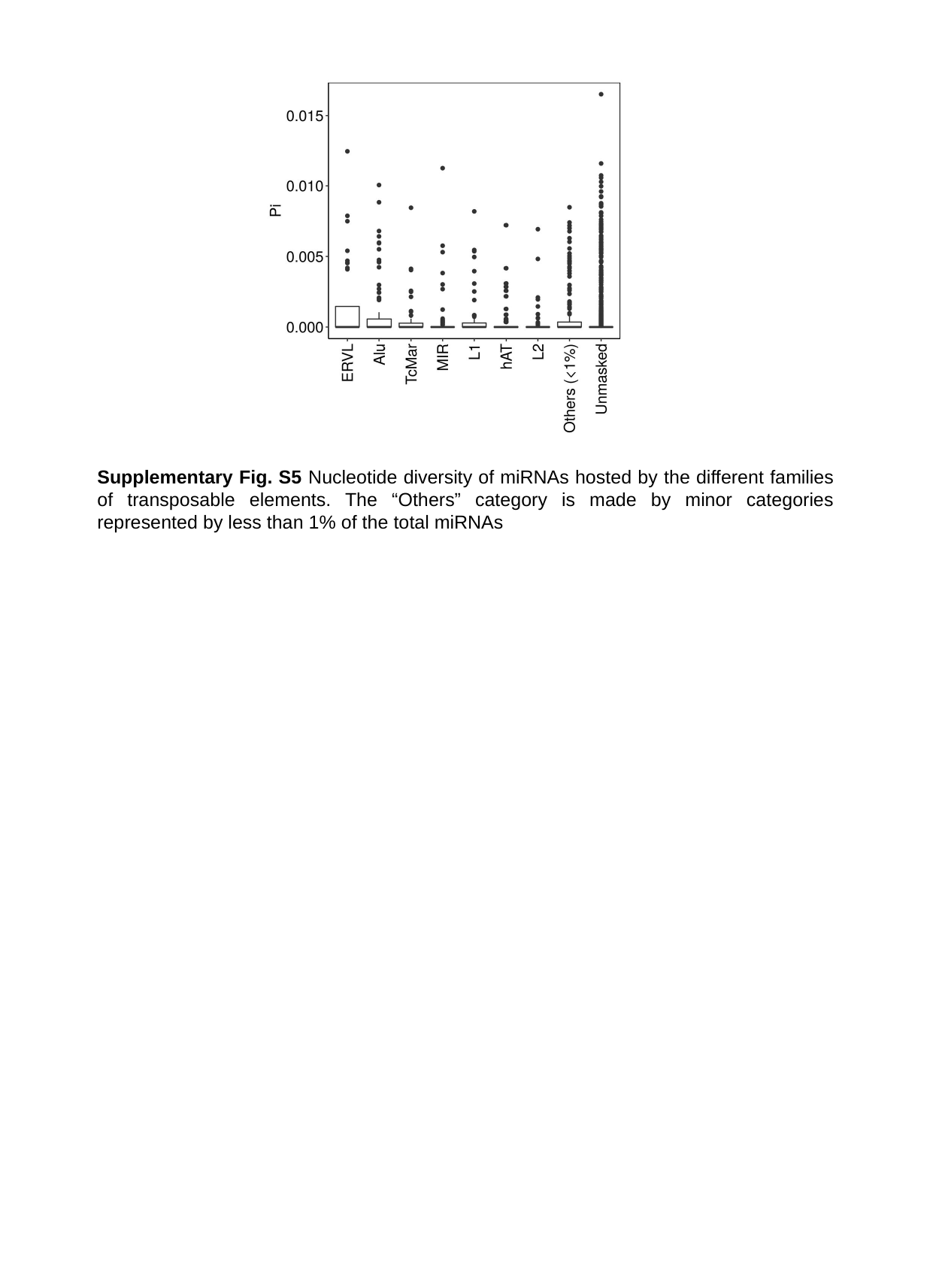

Supplementary Fig. S5 Nucleotide diversity of miRNAs hosted by the different families of transposable elements. The “Others” category is made by minor categories represented by less than 1% of the total miRNAs

## Slide 7
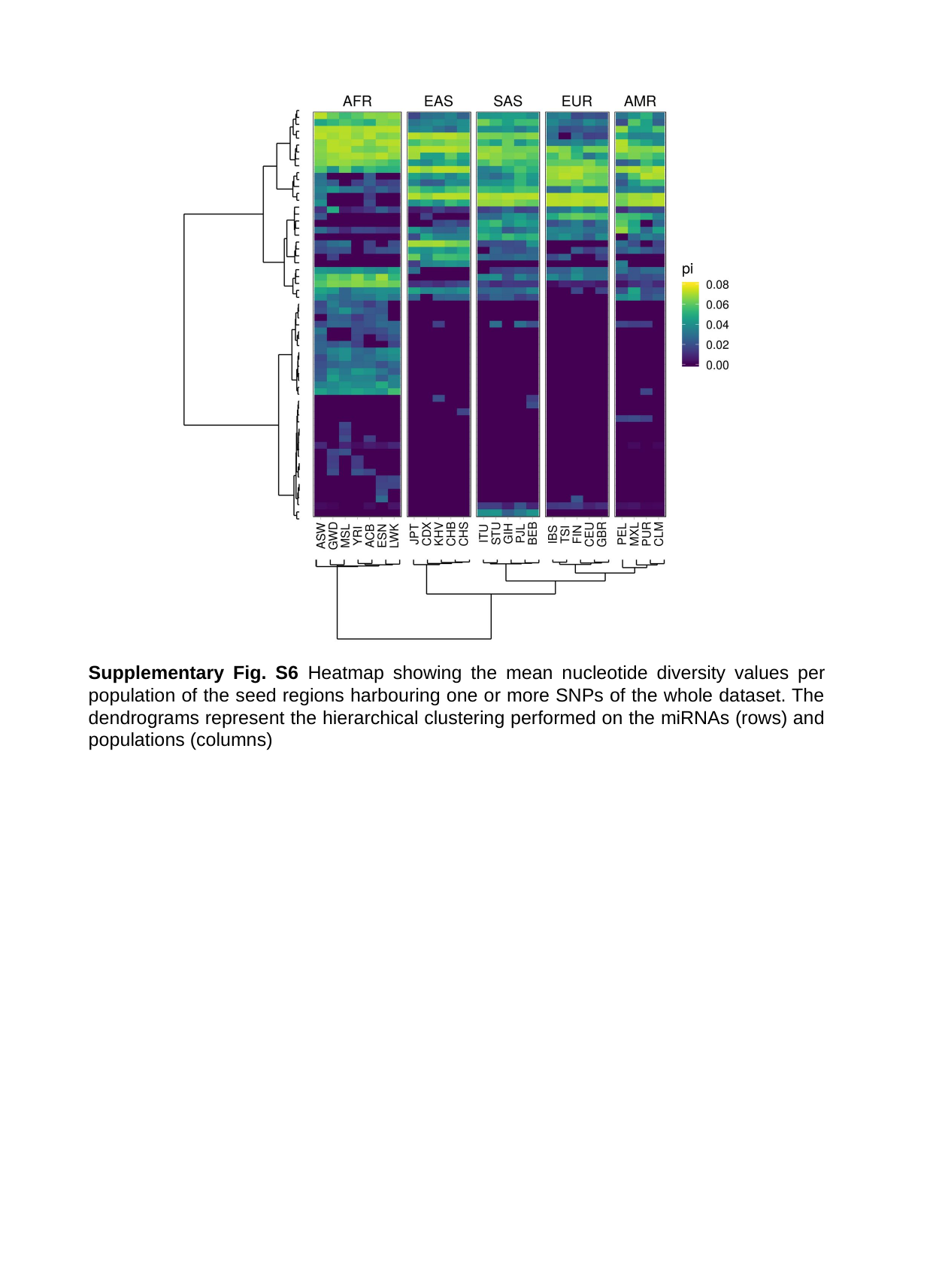

Supplementary Fig. S6 Heatmap showing the mean nucleotide diversity values per population of the seed regions harbouring one or more SNPs of the whole dataset. The dendrograms represent the hierarchical clustering performed on the miRNAs (rows) and populations (columns)
